# Effect of substituting hydroponic barley forage with or without enzymes on performance of growing rabbits

**DOI:** 10.1038/s41598-023-27911-x

**Published:** 2023-01-18

**Authors:** Ahmed A. A. Abdel-Wareth, Esraa M. H. Mohamed, Hamdy A. Hassan, Ahmed A. Eldeek, Jayant Lohakare

**Affiliations:** 1 Department of Animal and Poultry Production, Faculty of Agriculture, South Valley University, Qena, 83523 Egypt; 2 Poultry Production Department, Alexandria University, Alexandria, 21545 Egypt; 3 Poultry Center, Cooperative Agricultural Research Center, Prairie View A&M University, Prairie View, TX 77446 USA

**Keywords:** Animal physiology, Metabolomics, Developmental biology, Endocrinology, Gastroenterology, Physiology, Metabolism

## Abstract

This study aims to evaluate the effect of hydroponic barley (HB) by substituting control diet with 25% HB with or without enzymes on rabbit performance, nutrient digestibility, and economic efficiency. A total number of 60 growing male HyPlus rabbits (average body weight 669 ± 12 g, 30 days of age) were utilized in the present study. The rabbits were randomly assigned to three groups (n = 20 rabbits per group). The first group served as a control (C). The other two groups were fed the control diet substituted with 25% hydroponic barley HB (group CHB), and the control diet substituted with 25% HB added with 0.5 g/kg enzymes (CHBE). The experiment lasted for 56 days. The results revealed that daily body weight gain improved (P < 0.05) by 18.64% and 23.94%, and feed conversion ratio improved by 3.74% and 17.91% than control, respectively, during 30–86 days of age in CHB and CHBE groups. The economic efficiency was improved (P < 0.05) by 32.17% and 39.60% in CHB and CHBE diets, respectively, compared to control; and nutrient digestibility, and mineral retention of growing rabbits were also improved (P < 0.05) by substituting HB with or without enzymes compared to control diet. Overall, the best rabbit performances were observed in both CHB and CHBE groups. In conclusion, these results suggest that substituting 25% of concentrated control diet by hydroponic barley with or without enzymes have positive effects in a sustainable way on growth performance, nutrient digestibility, and economic efficiency of growing rabbits.

## Introduction

To increase economic profitability, the rabbit farmers will have to focus more on the sustainability of production and to look for cheap alternative feed sources. Rabbit meat production is becoming increasingly important around the world. Since feed costs account around 60–70% of total production cost, it is very important to feed the animals according to their nutrient requirements for potential production, and sustainability^1,2^. Hydroponic is now emerging as a novel alternative technology to grow fodder for the animal farm due to the limitations in the conventional method of cultivated green fodder in most of the Middle East and African countries^3–5^. Therefore, fodder is produced without using any soil, and growing the plants in water or mineral nutrient solution is known as hydroponics fodder or fresh fodder or sprouted grains^6,7^. Furthermore, the increase requirements of land for producing cereal grains have made traditional green fodder cultivation hard to be augmented, beside other constrains such as climate change, water deficiency or water salinity, growth period, fertilizer requisite and expenses^8,9^. This hydroponic technique can partly meet the growing requirement of animal feed with suitable prices, and assurance of a continuous green forage production of high quantity over the year. Hydroponic barley (HB) production is a high technique of growing barley in a clean environment without chemicals and artificial fertilizer^10^.

Furthermore, the biological and economic benefits of HB production and utilization depends on the agriculture conditions, dry matter (DM) content and water consumption^11,12^. The use of HB in feeding growing male or female rabbits showed significant nutritional benefits mainly due to its nutrients such as crude protein (CP) and ether extract (EE)^13^. Replacing HB at 20 or 40% of commercial feed in growing rabbit diets showed positive effects, however, higher level at 60% of HB in rabbit diets is not recommended because it negatively affects nutrients digestibility and feeding values of tested diets^10^.

Dietary enzymes supplementation can enhance the nutritive value of cereal grains and their by-products. Feeding growing rabbits on diets containing 15% yellow corn with enzymes and prebiotics improved nutrient digestibility of DM, CP, and EE in rabbits^14^. Previous studies produced a range of outcomes, which might be attributed to the growth period, environmental factors, and grown grain species. The results have encouraged a need for additional study on HB to determine the best dosage for rabbit diets. However, limited studies have focused on the use of supplementation of enzymes to HB diets to improve nutrient digestibility, animal health, particularly enhancing liver and kidney functions and supporting growth performances. Consequently, the idea is to use the xylanase and Beta-glucanase enzymes and HB in the rabbits’ diet to explore their synergistic effects in terms of maximizing the nutritional benefits. Therefore, this study aims to evaluate the substitution of a control diet with 25% HB with or without enzymes and their potential effects on productive performance, economic return, and nutrient digestibility in rabbits.

## Materials and methods

### Experimental animals, design, and management

Rabbits were housed at the rabbit research farm of the Department of Animal and Poultry Production, Faculty of Agriculture of the South Valley University, Qena, Egypt. Throughout the trial, the rabbits were handled in accordance with the principles for the care of experimental animals, and the experiment protocol (SVUAGR8/2018) was approved by the Committee of Ethics of the Animal and Poultry Production Department of the South Valley University. The study was carried out in compliance with the ARRIVE guidelines^15^. Rabbits were individually reared in cages (width × length × height: 50 cm × 60 cm × 40 cm) of galvanized wire net, equipped with manual drinker and feeder. The rabbits were reared in a closed house system with average temperature of 23 ± 2 °C with a 16 h light and 8 h dark during the experimental period. Fresh tap water was provided ad libitum through stainless steel nipples located inside each cage. During the experiment (56 days), the body condition and health of the rabbits were weekly inspected. A total of sixty male Hyplus rabbits, weaned at the age of 30 days (average body weight 669 ± 12 g), were utilized in the present study. The rabbits were randomly assigned to three groups of 20 rabbits each. The first group was fed only the control diet (Table 1) which was formulated to meet the energy and nutrient requirements of growing rabbits according to the recommendations of Lebas^16^. The other two groups were fed the control diet (C) substituted with 25% dried hydroponic barley (CHB) and the control diet substituted with 25% dried hydroponic barley with 0.5 g/kg enzymes (CHBE). The enzymes contained the Xylanase and Beta-glucanase (product name: AXTRA XB) for use in wheat and barley-based diets and diets containing high levels of dietary fibre (Danisco Animal Nutrition, Marlborough, Wilts, UK). In the substituted diets, the dried HB was substituted by removing 25% of the control diet, and then the whole diets including HB were pelleted without (CHB) or with enzymes (CHBE).

**Table 1:** Ingredient composition (as-fed basis) of the diet fed to rabbits throughout the experiment.

Ingredients, g/kg	
Corn	310
Wheat bran	200
Soybean meal (440 g/kg)	190
Wheat straw	120
Lucerne hay	50
Rice bran	50
Linseed straw	28
Sunflower meal	25
Limestone	20
Sodium chloride	3
Vitamin-mineral premix^a^	3
Dl-Methionine	1
Total	1000

^a^Per kg of diet: vitamin A 10,000 IU, vitamin D_3_ 900 IU, vitamin E 50.0 mg, vitamin K 2.0 mg, vitamin B_1_ 2.0 mg, folic acid 5.0 mg, pantothenic acid 20.0 mg, vitamin B_6_ 2.0 mg, choline 1200 mg, vitamin B_12_ 0.01 mg, niacin 50 mg, biotin 0.2 mg, Cu 0.1 mg, Fe 75.0 mg, Mn 8.5 mg, ZnO 20 mg.

### Hydroponic system of barely sprouting

A hydroponic system was constructed at the rabbit research farm, Department of Animal and Poultry Production, Faculty of Agriculture, South Valley University, Qena, Egypt. It consists of two units with metal frame each with dimensions (55 cm × 200 cm × 240 cm). Each unit consists of four shelves to hold 24 planting trays. Plastic trays with dimensions 90 × 30 × 4 cm were used for growing barley grains. Hydroponic system was kept at controlled temperature 24 ± 2 °C inside growth chamber and the relative humidity was between 65 to 70%. Clean barley seeds (*Hordeum vulgare* L.) were used to grow fodder by hydroponics technology in eight days. The cleaned seeds were soaked for 30 min in a 20% sodium hypochlorite solution. Planting trays were also cleaned and disinfected. The seeds were then washed well from residues of bleach and resoaked in tap water overnight (about 12 h) before planting. Seeds were germinated in the planting trays. Trays were irrigated manually, with tap water only twice a day (early in the morning and late in the afternoon). Fodder was harvested at day 8, separated manually using hands and then *sun*-*dried* before mixed with rabbit diets.

### Intake and growth performances

Rabbit's body weight (BW) values were recorded on d 30, 60 and 86 of life, respectively. Fresh feed was provided daily for ad libitum intake, and the residue was weighed the next day for the total experimental period of 56 days and daily feed intake was calculated. Feed conversion ratio (FCR) was calculated as feed per gain based on the weight of feed consumed divided by average daily body weight gain.

### Digestibility trial

Twenty animals were used in each treatment. For digestibility studies, the measurement period lasted four days from 84 to 88 days of age. During the four days, daily feed intake was determined, and total feces of the 20 replicates were collected, weighed, and kept in a freezer at − 10 °C until preparation for chemical analysis. Faecal samples were dried at 60 °C for 48 h (partial drying) before being analyzed.

Apparent nutrient digestibility of the nutrients was determined according to the following classical formula^17^:

$$\mathrm{Apparent \; nutrient \; digestibility }= \frac{NI-NE}{NI},$$

where NI is the nutrient intake and NE is the fecal nutrient excretion and mentioned as apparent nutrient digestibility coefficients.

### Chemical analysis

The diets, well-dried hydroponic barley and dried fecal samples were ground so that they can pass through a 1 mm screen using a grinder. Then, samples of the feed and feces were analyzed for moisture by oven drying (method No. 930.15), ash by incineration (method No. 942.05), protein by Kjeldahl (method No. 984.13), ether extract by Soxhlet fat analysis (method No. 954.02) and crude fiber (method No. 978.10) method described by the AOAC International^18^. Nitrogen-free extract (*NFE*) was calculated by subtractions. Digestible energy (DE) was evaluated according to Fekete^19^ using the following equation: DE (Kcal/kg) = (7.1 (CP, g/kg)) + (12 (EE, g/kg)) + (5.59 (NFE, g/kg)). The minerals like Mg, P, K, Ca, Mn, Fe, and Zn contents in the diets and feces were measured (Element analyzer with energy dispersive X-ray fluorescence system, JSX 3222, JEOL, Japan).

### Economic efficiency

Economic efficiency was calculated according to Raya et al.^20^ from the following equation:

$$ {\text{Economic efficiency }}\left( \% \right) \, = \, \left( {{\text{Net revenue}}/{\text{ Total feed cost}}} \right) \times {1}00 $$

where;

$$ {\text{Net revenue }} = {\text{ Price of weight gain }} - {\text{ Total feed cost}}. $$

$$ {\text{Price of weight gain }} = {\text{ Average weight gain }}\left( {{\text{kg}}/{\text{head}}} \right) \, \times {\text{ Price}}/{\text{kg live BW}}. $$

$$ {\text{Total feed cost }} = {\text{ Average feed consumption }}\left( {{\text{kg}}/{\text{head}}} \right) \, \times {\text{ price }}/{\text{kg feed}}. $$

### Statistical analysis

Data were subjected to one-way analysis of variance (ANOVA) using the general linear model procedure of SAS/STAT 9.2 User’s Guide (SAS Institute^21^):

$$ {\text{Y}}_{{{\text{ik}}}} = {\text{ U }} + {\text{T}}_{{\text{i}}} + {\text{ E}}_{{{\text{ijk}}}} $$

where, Y_ik_ = observed value of the response variable, U = observed mean for the response variable, T_i_ = the fixed effect of treatment group, E_ijk_ = random error.

The differences among the means of individual treatments were tested with Duncan’s multiple range tests (Duncan^22^). Significance was declared at *P* ≤ 0.05: *P* values less than 0.001 were expressed as “ < 0.001” rather than the actual value.

### Ethics approval and consent to participate

The Institutional Animal Care and Use Committee of South Valley University approved the experiment protocol, and the study was carried out according to the guidelines of the Egyptian Research Ethics Committee and the guidelines in the Guide for the Care and Use of Laboratory Animals (2011).

### Institutional review board statement

The procedures used were approved by the Authors' institution (Department of Animal and Poultry Production, South Valley University, Egypt) Ethics Committee (SVUAGR8/2018), and care was taken to minimize the number of animals used.

## Results

### Chemical composition of diet and hydroponic barely

The DM, CP, CF, EE, and calculated DE contents of HB were similar in nutritive value to the control diet (Tables 2 and 3). Interestingly, the CP was 18.22, 18.06, 18.10 and 17.84% in Control, CHB, CHBE and HB, respectively. Also, EE was 4.33, 4.35, 4.65 and 4.36% in Control, CHB, CHBE and HB, respectively. The control diet, CHB, CHBE and HB resulted in 12.28, 13.34, 12.76 and 10.82% of CF, respectively. Furthermore, DE (MJ/kg DM) was 18.65, 18.66, 18.64 and 19.82 in Control, CHB, CHBE and HB, respectively. The Fe and P were higher in CHB, CHBE and HB than the control diet.

**Table 2:** Chemical analysis of the experimental diets.

Items %	Control	CHB	CHBE
Dry matter	96.11	95.75	96.07
Crude protein	18.27	18.06	18.10
Ether extract	4.33	4.35	4.69
Nitrogen free extract	54.53	53.87	53.52
Crude fiber	12.28	13.34	12.76
Digestible energy (MJ/kg DM)^a^	9.35	9.47	9.45
Mg	1.92	0.21	0.18
Al	0.00	0.07	0.07
Si	0.15	0.33	0.34
P	0.56	0.75	0.82
S	0.27	0.17	0.20
K	2.37	2.22	2.11
Ca	6.22	5.80	5.86
Mn	0.02	0.02	0.02
Fe	0.16	0.34	0.32
Zn	0.02	0.02	0.02

HB: hydroponic barley; CHB: Control diet substituted with 25% hydroponic barley; CHBE: Control diet substituted with 25% hydroponic barley with 0.5 g/kg enzymes.

^a^DE estimated according to Fekete^19^.

**Table 3:** Chemical analysis of hydroponic barley.

Nutrients	Proportion (%)	Ash elements	Proportion (%)
Dry matter	16.87	P	1.47
Crude protein	17.84	Ca	2.74
Ether extract	4.36	Mn	0.03
Nitrogen free extract	62.62	Fe	1.03
Crude fiber	10.82	Zn	0.06
Digestible energy (MJ/kg DM)^a^	9.92	Mg	0.26

^a^DE estimated according to Fekete^19^.

### Growth performance

The results showed that the growing rabbits fed on CHB and CHBE diets recorded the highest body weight and daily body weight gain (P < 0.05) compared to the control group at 58 and 86 days of age (Table 4). Interestingly, dietary CHB and CHBE increased daily body weight gain (P < 0.01) by 18.64% and 23.94% than control, respectively, during 30–86 days of age. Likewise, the rabbits fed on CHB and CHBE diets had the best (P < 0.01) feed conversion ratio in comparison to the control during 30–58 and 30–86 days of age (Table 4). The dietary CHB and CHBE improved feed conversion ratio (P < 0.01) by 3.74% and 17.91% than control, respectively during 30–86 days of age. On the other hand, feed intake showed no differences among the treatments.

**Table 4:** Effects of hydroponic barley with or without enzymes on growth performance of growing rabbits.

Items	Treatments			SEM	P-Value
	Control	CHB	CHBE		
Body weight, g					
30 days of age	668	652	689	16	0.649
58 days of age	1277^b^	1406^a^	1469^a^	29	0.015
86 days of age	1946^b^	2166^a^	2273^a^	39	0.001
Daily weight gain, g					
30–58 days of age	21.75^b^	26.93^a^	27.86^a^	2.56	0.008
58–86 days of age	23.89^b^	27.14^a^	28.71^a^	2.46	0.019
30–86 days of age	22.82^b^	27.04^a^	28.29^a^	2.67	0.001
Daily feed intake, g					
30–58 days of age	76.93	79.75	80.14	4.16	0.704
58–86 days of age	100.71	105.64	106.68	3.89	0.182
30–86 days of age	88.82	92.70	93.41	6.44	0.222
Feed conversion ratio					
30–58 days of age	3.66^a^	2.98^b^	2.92^b^	0.13	0.026
58–86 days of age	4.21^a^	3.89^b^	3.72^b^	0.12	0.023
30–86 days of age	3.82^a^	3.45^b^	3.32^b^	0.08	0.004

SEM: Standard error of the means (n = 20); CHB: Control diet substituted with 25% hydroponic barley; CHBE: Control diet substituted with 25% hydroponic barley with 0.5 g/kg enzymes.

^a,b^Means within the same row bearing different superscripts differ significantly (P < 0.05).

### Nutrient digestibility coefficients

Digestibility of nutrients and energy of growing rabbits are shown in Table 5. Feeding CHB with or without enzymes supplementation to growing rabbits significantly increased (P < 0.01) the digestibility of DM, OM, CP, CF and EE when compared to control diet (Table 5). Interestingly, dietary CHB and CHBE enhanced the digestibility of CP by 5.32% and 6.80%, respectively, compared to control (P < 0.002). In comparison to the control, the dietary CHB and CHBE improved the digestibility of EE by 4.84% and 8.83%, respectively (P < 0.002). Moreover, providing CHB or CHBE diets improved (P < 0.002) the digestible energy content compared to control. Dietary CHB and CHBE enhanced (P < 0.002) energy digestibility by 3.17% and 3.93%, respectively, when compared to the control. Similarly, the digestibility of Ca, Mn, and Zn were higher (P < 0.05) in CHB with or without enzymes in comparison with the control diet, however, Mg, K and P digestibility were greater in CHBE than the CHB or control diet (Table 6).

**Table 5:** Effects of hydroponic barley with or without enzymes on the nutrient digestibility coefficient of growing rabbits.

Items	Treatments			SEM	P-value
	CHB	CHBE	CHB		
Dry matter	0.632^b^	0.655^a^	0.661^a^	0.005	0.020
Organic matter	0.621^b^	0.649^a^	0.659^a^	0.005	0.002
Crude protein	0.676^b^	0.712^a^	0.722^a^	0.006	0.002
Ether extract	0.668^b^	0.701^a^	0.727^a^	0.008	0.006
Crude fiber	0.415^b^	0.477^a^	0.471^a^	0.008	0.013
Nitrogen free extract	0.643^b^	0.669^a^	0.676^a^	0.005	0.009

SEM: Standard error of the means (n = 20); CHB: Control diet substituted with 25% hydroponic barley; CHBE: Control diet substituted with 25% hydroponic barley with 0.5 g/kg enzymes.

^a,b^Means within the same row bearing different superscripts differ significantly (P < 0.05).

**Table 6:** Effects of hydroponic barley with or without enzymes on mineral digestibility coefficient of growing rabbits.

Items	Treatments			SEM	P-value
	Control	CHB	CHBE		
Mg	0.438^b^	0.442^b^	0.467^a^	0.005	0.028
P	0.333^c^	0.381^b^	0.452^a^	0.012	0.001
K	0.432^b^	0.442^b^	0.465^a^	0.005	0.025
Ca	0.535^b^	0.557^a^	0.564^a^	0.005	0.020
Mn	0.538^b^	0.640^a^	0.654^a^	0.021	0.013
Fe	0.217	0.231	0.242	0.005	0.132
Zn	0.381^b^	0.424^a^	0.435^a^	0.008	0.012

SEM: Standard error of the means (n = 20); CHB: Control diet substituted with 25% hydroponic barley; CHBE: Control diet substituted with 25% hydroponic barley with 0.5 g/kg enzymes.

^a–^^c^Means within the same row bearing different superscripts differ significantly (P < 0.05).

### Feed cost and economic return

The cost of total feed intake was decreased by 7.39% and 1.41% in substituting control diet by 25% CHB and CHBE, respectively. This decrease was mainly due to the lower price of 1 kg of diet containing 25% HB compared with the control diet (Table 7). The daily weight gain and so selling price were increased in the rabbits fed diet containing 25% HB. In addition, the highest value of net revenue was obtained in the rabbits fed diets with 25% HB, compared to the control diet. Moreover, rabbits fed diets with CHB and CHBE showed better economic efficiency than the control diet. Dietary CHB and CHBE increased economic efficiency by 32.17% and 39.60%, respectively, as compared to the control.

**Table 7:** Effects of hydroponic barley with or without enzymes on economic return of growing rabbits.

Items	Treatments			SEM	P-value
	Control	CHB	CHBE		
BWG, g	1278^b^	1514^a^	1584^a^	39.5	0.001
Feed intake, g	4974	5191	5231	64	0.222
Price BWG (EGP)	74.2^b^	83.94^a^	88.38^a^	7.671	0.001
Cost of feed (EGP)	24.64^a^	22.86^b^	23.18^b^	1.240	0.003
Net revenue (EGP)	49.56^b^	61.08^a^	65.26^a^	5.237	0.001
Economic efficiency (%)	202^b^	267^a^	282^a^	24.1	0.001

SEM: Standard error of the means (n = 20); CHB: Control diet substituted with 25% hydroponic barley; CHBE: Control diet substituted with 25% hydroponic barley with 0.5 g/kg enzymes; BWG: Body weight gain; EGP: Egyptian pounds.

1 USD = 15 EGP.

$$\mathrm{Economic \; efficiency }(\mathrm{\%}) =\frac{\mathrm{Net  \; revenue }}{\mathrm{Total  \; feed  \; cost}} \times 100$$.

Net revenue = Price of weight gain − total feed cost.

^a,b^Means within the same row bearing different superscripts differ significantly (P < 0.05).

## Discussion

Evaluating the beneficial effects of substituting HB in rabbit feed is important aspect for the sustainability of products and productivity in rabbit production. In the current study, the DM, DE, CP, CF, EE and minerals in HB were almost comparable with the nutrients in the control diet. It is known that the contents of energy and protein in the diets of growing rabbits affect their growth rate. Therefore, it was necessary to determine the chemical composition of HB prior to its use as it may provide useful information about its effect on rabbits. As reported by Abouelezz and Hussein^13^, the use of HB in feeding growing male or female rabbits showed significant improvements in growth performances mainly due to HB nutritional contents. The composition of HB used in the present study was consistent with those reported in the literature. For instance, barley seed contained 93.1% DM, 97.6% OM, 12.7% CP, 6.3% CF and 1.8% EE (Mehrez et al.)^23^, and it contained 93.81% DM, 11.11% CP, 8.9% CF and 1.68% EE according to Gebremedhin et al.^9^ Also noted by Kide and Abrha^12^ was the presence of 11.11% CP, 3.35% EE and 8.9% CF in HB cultivated for 6 days. Sprouting of cereal grains produced an increased nutrient quantity and quality such as protein, digestible energy, fats and minerals contents^24^. Based on the earlier research, it was determined that the nutrient composition of HB, including DM, OM, CP, CF and EE were suitable for partially substituting the concentrate feed in rabbits. Likewise, Abouelezz et al.^25^ indicated that HB contains 23.3% CP, 4.17% EE, 26.7% NFE, and 3.97% ash on DM basis, reportedly higher values than other studies. A change in the weight of any one of the nutrients also caused equivalent changes in the compositions of the other nutrients. The chemical composition of HB ranged from 14–17.5% DM, 13.5 to 23.5% CP, 11.4 to 15.9% CF, 3.2 to 5.67% EE, 25.5 to 35.5%^9,10,12–14,14,15,25^ showing the nutritive content of HB in different studies are in line with the nutrient contents observed in our study. Some variations in the nutritive value of HB in the previous studies may be due to HB growth period, cultivation conditions as well as the chemical methods.

The current study revealed that the growing rabbits fed on CHB and CHBE diets recorded the highest daily body weight and daily body weight gain (P < 0.05) compared to the control group during 58 and 86 days of age. Similarly, the CHB and CHBE diet-fed rabbits exhibited the best feed conversion ratio compared to the control group. These findings are in agreement with Abouelezz and Hussein^13^ who found that the male rabbits fed on HB recorded the highest body weight, body weight gain and feed intake compared to the control group. The substitution of corn with 20% of barley grains in the diet of growing male New Zealand White rabbits had significantly higher (P < 0.05) live body weight and the best FCR^14^. According to Gabr et al.^10^, feeding growing male rabbits HB by replacing some of their concentrate feed mixture diet led to enhanced body weight gain, relative growth rate, and performance index compared to the rabbits fed the control diet. Conversely, feeding growing rabbits 60% HB pellets in place of commercial feed for 32 days reduced their feed intake and growth rate in comparison to the control group^15^. On the other hand, some researchers indicated that replacing pelleted commercial feed by HB has negative effects on growth performances^26–28^.

In the current study, synergistic effects of using commercial enzymes and HB in the rabbits' diet and using simply HB were examined in order to maximize the productive performance. There is no information available on addition of enzymes to HB substituted diets in rabbits. Therefore, additional studies that used enzymes in rabbit diets were used in order to compare our findings. El-Aziz et al.^29^ reported that supplementation of rabbit diets with a mixture of enzymes and sodium butyrate at 0.5 g/kg diet significantly (P < 0.05) improved growth performance than the control group. On the other hand, dietary treatments consisting of supplementation of 0, 35, 50, and 70 g Natuzyme plus/100 kg concentrate mixture for weaned crossbred New Zealand White rabbits had no significant (P > 0.05) differences in daily live weight gain and feed conversion ratio for the tested rabbits compared to control^30^. Furthermore, supplementation of enzymes preparation containing cellulase, α-glucanase, α-amylase, protease and lipase to New Zealand White rabbit significantly improved body weight gain, relative growth rate and feed conversion ratio^31^. Likewise, Shanmuganathan et al.^32^ found that supplementation of rabbit diets with the exogenous enzymes (cellulases and proteases), yeast culture or probiotic showed highest daily feed intake, daily body weight gain and the lowest feed/gain ratio. In the current study, the nutrient digestibility was increased (P < 0.05) when growing rabbits were fed CHB with or without enzyme supplementation as compared to control. Additionally, either CHB or CHBE increased the ability to digest energy compared to control. Similarly, the mineral digestibility was improved in CHB with or without enzymes in comparison with the control diet. Additionally, HB is high in enzymes; consequently, feeding HB promotes animal productivity and digestibility by removing the acidic conditions^23^. The digestibility of OM, CP, and NFE in HB were higher than the values of control diet^29^. Substitution of concentrate diet by fresh 8 days-age HB at 25, and 50% with or without probiotics to growing male New Zealand white rabbits improved the digestibility of DM, CP, CF and NFE compared to concentrate diet^27^. In contrast, values of CP and CF digestibility for the group fed the 20% HB diet were significantly higher than those fed the 40% HB diet and control diet, according to Mehrez et al.^23^ showing higher levels have detrimental effects on nutrient digestibility. The improvements in digestibility may be attributable to a large proportion of leafy, root sprouts, which the gut microflora enzymes can easily digest and hydrolyze, as well as enzymatic digestion, like the proteases found in plant cells' lytic vacuoles. Similarly, supplementation of enzymes to rabbit diets improved the digestibility of DM, CP, CF, and energy^32^. However, the dietary multi-enzymes (Natuzyme plus) treatments at different levels did not show statistically significant differences (P > 0.05) in digestibility of DM, CP, and EE but the CF and NFE digestibility was improved in the rabbits fed diet supplemented with 50 g enzymes/100 kg diet^30^. Likewise, addition of enzymes in diets of New Zealand White rabbits significantly improved digestibility of nutrients compared with those without enzymes addition^31^. The enzymes can partially hydrolyze non-soluble protein, reduce the viscosity of gut contents, and result in improvements in nutrient absorption and therefore improved growth performance of rabbits.

By replacing the control diet with 25% CHB or 25% CHBE, the cost of total feed was dropped by 7.39 and 1.41%, respectively. This decrease was mainly due to the lower price of 1 kg of diet containing 25% HB compared with the control diet. The body weight gain and selling price were increased in the rabbits fed diet containing 25% HB. In addition, the highest value of net revenue was obtained in the rabbits fed diet with 25% HB, while the lowest value was observed for those fed control diet. In comparison to control groups, rabbits fed diets containing 25% HB demonstrated the highest economic efficiency. Nutritionists' primary goals in recent years have been to raise rabbit performance and lower feeding expenses. Moreover, the most important factor in achieving the maximum meat production efficiency values depend on the body weight gain, the growing period length, and the cost of feed. Application of HB as a substitute for concentrate feed in growing rabbit diets improved the economic returns and reduced the total feed cost^28^. Abouelezz and Hussein^13^ reported that substituting HB to the commercial diet of fattening rabbits reduced feed cost per gain than the control. As economic standpoint, HB has a short growth period of 7–10 days and requires a small piece of land area for production^10^.

In the current study, using a combination of commercial enzymes and HB in the rabbits’ diet showed positive effects compared to control group in terms of maximizing the profitability. Dietary supplementation of multi-enzymes with sodium butyrate is highly recommended in growing rabbits due to their beneficial effects on the growth performance and profitability^32^.

## Conclusions

In conclusion, the present results suggest that substitution of 25% of the concentrate feed by hydroponic barley with or without enzymes in the growing rabbit’s diet improved the growth performance, nutrient digestibility, and economic efficiency. HB is now emerging as a novel alternative feedstuff for sustainable rabbit production. There is no practical need to add the enzymes as it does not show any additional benefits compared to CHB. Nevertheless, further studies are needed to explore the mechanism of action of HB on the beneficial response in growing rabbits.

## Data Availability

All data generated and analyzed in the present study are available from the corresponding author on reasonable request.
